# Protein complex prediction using Rosetta, AlphaFold, and mass spectrometry covalent labeling

**DOI:** 10.1038/s41467-022-35593-8

**Published:** 2022-12-21

**Authors:** Zachary C. Drake, Justin T. Seffernick, Steffen Lindert

**Affiliations:** Department of Chemistry and Biochemistry, Ohio State University, Columbus, OH 43210 US

**Keywords:** Protein structure predictions, Computational biophysics, Mass spectrometry

## Abstract

Covalent labeling (CL) in combination with mass spectrometry can be used as an analytical tool to study and determine structural properties of protein-protein complexes. However, data from these experiments is sparse and does not unambiguously elucidate protein structure. Thus, computational algorithms are needed to deduce structure from the CL data. In this work, we present a hybrid method that combines models of protein complex subunits generated with AlphaFold with differential CL data via a CL-guided protein-protein docking in Rosetta. In a benchmark set, the RMSD (root-mean-square deviation) of the best-scoring models was below 3.6 Å for 5/5 complexes with inclusion of CL data, whereas the same quality was only achieved for 1/5 complexes without CL data. This study suggests that our integrated approach can successfully use data obtained from CL experiments to distinguish between nativelike and non-nativelike models.

## Abstract (web summary)

Covalent labeling (CL) from mass spectrometry experiments provides structural information of higher-order protein structure. Here, the authors develop an algorithm which integrates experimental CL data to predict protein complexes in the Rosetta molecular modeling suite using AlphaFold models.

## Introduction

Mass spectrometry (MS) is a versatile analytical approach which has become a vital tool in structural biology, capable of probing the structure and dynamics of protein assemblies^1,2^. Protein-protein complexes are central in many crucial biological and cellular processes^3^, which makes their structural elucidation important. Currently, over 182,000 protein structures have been determined and archived in the Protein Data Bank (PDB), with around 114,000 of these being protein-protein complexes^4^. These high-resolution protein structures can be obtained using techniques such as nuclear magnetic resonance (NMR)^5^, cryo-electron microscopy (cryo-EM)^6^, and most notably X-ray crystallography^7^. However, structures at atomic resolution are not always obtainable due to limitations of the forementioned techniques in areas such as acceptable system size, required sample concentration, and excessive sample conformational heterogeneity.

Structural mass spectrometry is an alternative method which generally requires less time for sample preparation, can handle smaller sample sizes, is usable for a large range of protein sizes, and provides sparse biophysical data that can be used to gain insight into a variety of protein structural characteristics. Although MS experiments cannot comprehensively determine a high-resolution protein structure, insights into conformational states can be obtained, validation of existing models can be achieved, or the data can be supplemented with computational techniques for atomic-detail structure elucidation. A few common approaches in structural MS are chemical cross-linking^8^, hydrogen-deuterium exchange (HDX)^9,10^, surface-induced dissociation (SID)^11,12^, ion mobility (IM)^13^, and covalent labeling of macromolecules (CL)^14,15^. Chemical cross-linking involves using chemical reagents that form covalent bonds to link specific functional groups within or across protein molecules, providing distance restraints. HDX methods can be used to study protein structure and dynamics using exchange between protein backbone amide protons and deuterium atoms from solution, which is sensitive to local solvent accessibility and flexibility. SID methods involve the soft ionization of native protein complexes into the gas phase which are then collided with a rigid surface where they can break apart into monomers or other intact subcomplexes. This method can provide information regarding the stoichiometry, connectivity, and interface strength of complexes. IM can offer structural information regarding the shape and size of a protein complex by analyzing the travel of a protein through a bath gas, providing an averaged cross-sectional area of the system. Finally, covalent labeling probes protein structure by exposing solvent-accessible amino acid side chains with either specific or nonspecific reagents that covalently bind. Differences in reactivity to labeling agents can distinguish between exposed and buried residues, as well as residues located at the surface of interacting domains in the case of protein complexes.

Covalent labeling offers several advantages over other MS techniques. For example, the challenging low abundance of specific interpeptide cross-links and complicated tandem MS fragmentation of chemically cross-linked peptides are not an issue for covalent labeling techniques^16^. Furthermore, due to the formation of stable, covalent bonds, the labeling of amino acids with labeling reagents are usually irreversible, unlike HDX where back-exchange frequently occurs, adding additional layers of complexity. Sparse structural data can be obtained from covalent labeling experiments with reagents such as hydroxyl radicals, carbenes, trifluoromethylations (CF_3_), diethylpyrocarbonate (DEPC), and sulfo-N-hydroxysuccinimide acetate (NHSA)^17^. These experiments provide metrics of modification that depend on the reactivity, solvent accessibility and potentially other structural features of the specific residues in solution. Structures of protein complexes can be further probed by comparing the degree of modification in the unbound and bound states, when possible. Interface residues are generally identified by examining large changes in modification rates between the unbound/bound state of a complex as solvent accessibility is likely to most dramatically change at the protein-protein interfaces. For example, a residue that gets labeled readily in the monomer, but not in the complex is likely part of the interface; although protein-protein binding could cause tertiary conformational changes, which might also result in changes in modification. The data obtained through a covalent labeling MS experiment can thus be used to probe higher orders structure of protein complexes.

As an alternative to experimental methods, modern computational methods have seen great success in accurately predicting and modeling protein tertiary structure^18,19^. The recent release of AlphaFold2^20^ (AF2, from DeepMind) has resulted in a revolution in the accuracy of computational protein modeling. AlphaFold^21^ is a neural network-based model that takes advantage of sequence coevolution data which has shown remarkable success and has outperformed other prediction methods during the 13th and 14th (with AF2) Critical Assessment of Techniques for Protein Structure Prediction (CASP)^22,23^, a series of blind tests to gauge the current state of protein structure prediction. AlphaFold-Multimer^24^ was released in 2021 and uses the AF2 model but was trained to predict multimeric complexes from sequences of multiple chains. Similarly, traditional protein-protein docking algorithms are useful for analyzing and predicting models of complexes. In protein-protein docking, monomeric structures (which can be obtained in a variety of ways) are used as input, and structures of the complex are predicted, with favorable orientations of the different subunits. Existing protein-protein docking algorithms which have been successful include ClusPro^25^, HDOCK^26^, ZDOCK^27^, SwarmDock^28^, HADDOCK^29^, PIPER^30^, and RosettaDock^31^. RosettaDock is a part of the Rosetta^32^ molecular modeling software suite which contains a large variety of algorithms for computational modeling and analysis of protein structures.

Incorporation of sparse experimental data into algorithms predicting protein structure can further improve computational predictions^33–35^. Information obtained from hydroxyl radical footprinting (HRF), HDX, and DEPC labeling experiments have been shown to improve tertiary structure prediction with Rosetta^36–43^ by using calculated solvent exposure metrics for models to select for experimentally accurate predictions. Similarly, protein shape and size information obtained through collisional cross-section data from IM experiments has also improved Rosetta structure prediction^44^. A method iSPOT^45^, which uses a combination of multiple biophysical methods (integration of shape information from small-angle X-ray scattering and protection factors probed by hydroxyl radicals), has been shown as a powerful approach for integrated modeling of multiprotein complexes. Isotope exchange using HDX-MS has been used to improve protein complex prediction by simulating complex isotope patterns and comparing to those obtained experimentally^46^. Similarly, differential HDX data has been incorporated into protein-protein docking to study the human uracil-DNA-gycosylase (hUNG) and its protein inhibitor (UGI)^47^. The use of differential covalent labeling has yet to be implemented within the RosettaDock framework. Although AF2 has proven to be an excellent and revolutionary method of protein structure prediction, there remain limitations, particularly for protein complexes^48^. Covalent labeling has the potential to help overcome some of these limitations and Rosetta is uniquely suited for the development of hybrid methods incorporating labeling data as additional scoring terms, for which there are many examples^36,39,41,49,50^. Here, we use RosettaDock to assemble protein complex subunits that were generated using AF2 and employ covalent labeling data to improve protein complex structure prediction.

In this study, we develop the computational framework (Supplementary Fig. 1) for using covalent labeling data in protein complex modeling in cases when state-of-the-art methods (both AlphaFold-Multimer and Rosetta) underperform. We propose a score term dependent on differential covalent labeling data obtained from HRF, DEPC, or NHSA experiments which when combined with the Rosetta score function readily selects computational models which agree with experimentally determined structures. We first observe a correlation between differential modification rates and inter-subunit residue distances within a protein complex based on our structural hypothesis that interface residues will see greater changes in solvent accessibility upon complex formation. Next, we develop a protocol where AF2 was used to generate structures of the protein subunits which were used as input for docking simulations. In a benchmark of 5 complexes, inclusion of our score term predict 5/5 structures with root-mean-square deviation (RMSD) less than 3.6 Å when compared to the native crystal structure, as opposed to 1/5 without CL data.

## Results and discussion

### Correlation of differential covalent labeling and residue proximity to binding interface

We hypothesized that differential covalent labeling data could be used to determine which residues are likely to be located at the binding interface within a protein complex. Surface residues can participate in molecular interactions with nearby solvent molecules. If these residues are located at the binding interface when part of a complex, upon binding, the side chains of these residues become buried and only interact with the neighboring residues of an adjacent bound subunit, decreasing the number of solvent interactions and the probability of that residue being labeled. In this case, one would expect to observe large changes in the frequency of modification for interface residues between the unbound and bound states of complexes due to large changes in solvent accessibilities in these regions. Based on this hypothesis, large decreases in modification of residues from the unbound to bound state of a complex have a higher probability of participating in the interface.

**Fig. 1:**
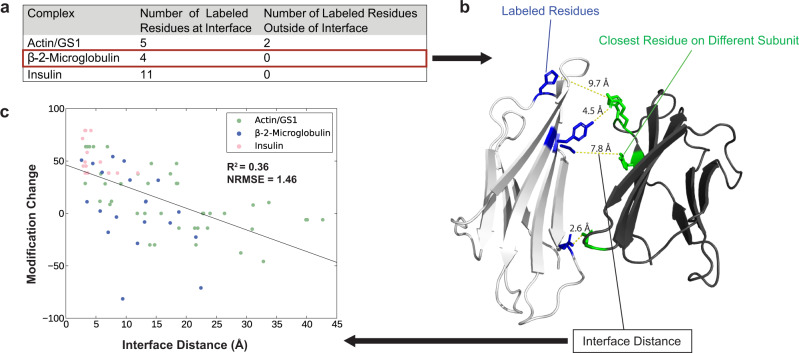
Quantifying the relationship between modification change and proximity to interface. **a** Breakdown of the residues with a modification change of at least 40%, within and outside the binding interfaces of a specified complex. The residues shown in Panel (a) are a subset of the residues shown in Panel **c**. **b** Visualization of the four labeled residues with large modification changes (see Panel **a**) for one subunit of the β−2-microglobulin homodimer crystal structure. The labeled residues are colored in blue, the respective closest residue on a different subunit of the complex is colored in green, and the interface distance between them is shown above the dotted yellow line. **c** Linear correlation between modification changes of all labeled residues and the interface distances, where larger modification changes are expected for residues at the interface (x = 0). Source data are provided as a Source Data file.

To test the proposed hypotheses, we used a benchmark set of protein complexes with publicly accessible differential labeling data and a native crystal structure. This dataset consisted of actin bound to gelsolin segment 1 (actin/gs1, heterodimer, PDB ID: 1YAG)^51^, β−2-microglobulin (homodimer, PDB ID: 2F8O)^52^, and insulin (hexamer of heterodimers, PDB ID: 4INS)^53^. To establish the validity of the hypothesized relationship with experimental data, the native crystal structures of these complexes were used to analyze the proximity of residues (using inter-residue distance) to the interface as a function of modification rate in the monomer compared to the complex. We assumed large-scale conformational changes do not occur upon complex formation after examining all subunits of each complex which contained labeled residues and finding that the average RMSD of the unbound (Actin PDB ID: 3HBT^54^, β-2-microglobulin PDB ID: 2D4F^55^, and Insulin PDB ID: 3I40^56^) to the bound subunit crystal structures was 2.1 Å. To quantify the amount of change in modification occurring between the unbound/bound state, a modification change was calculated from modification rates/extents of each state (see methods for full detail) with a larger positive value indicating a more significant decrease in modification from the unbound to bound state. We hypothesized that such a large decrease in modification from the unbound to bound state would likely be indicative of residues at the interface. The maximum modification change for a labeled residue observed across all three complexes was 80% and the average change was 18%. To isolate residues with large modification changes, we considered only residues that saw at least a 40% change in modification between unbound/bound states. Residues that were within 10 Å of the other chain were considered a part of the binding interface, which is consistent with the interface definition for the iRMSD calculations using DockQ^57^. From a total of 78 labeled residues across all complexes in the benchmark set, 38 of these residues were at the interface (distance < = 10 Å), and 40 of these residues were outside the interface (distance > 10 Å). The average modification percentage of residues in the interface was 38.59%, while the average modification percentage was -0.04% for residues outside the interface. We first used this criterion to compare the native structures to experimental data. Figure 1a lists the number of labeled residues with a modification percentage greater than or equal to 40% at and outside the interface and those residues are visualized for β-2-microglobulin (Fig. 1b). For all three complexes, the majority of labeled residues with modification changes greater than or equal to 40% were found to be located at the binding interface of the complex, with all the designated residues being at the interface for two of the complexes. The two exceptions were residues P322 and M325 located on a connecting loop region between two α-helices on the actin portion of the actin/gs1 complex, near the interface. Their peripheral locations to the interface may be the cause for the observed large modification changes, or those may be due to local structural changes which may result upon binding to gs1. Previous work showed that error present in covalent labeling data is acceptable up to a maximum of 35% of surfaced exposed residues being incorrectly identified as false negatives and 10% of buried residues identified as false positives while still providing accurate protein structure prediction^37^. In our benchmark, 91% of labeled residues with modification changes greater than or equal to 40% were close to protein-protein interfaces, resulting in a false positive rate of 9% which was within acceptable tolerances. This small preliminary analysis supported our hypotheses and indicated covalent labeling can be used to distinguish particular interface residues based off large changes in labeling.

Furthermore, we hypothesized that a larger distance to the binding interface for a particular residue would result in less solvent accessibility change when comparing unbound and bound states. For this reason, we would expect a smaller change in modification between the unbound/bound states of a complex. Combining all labeling data from all three complexes along with the interface distances of these labeled residues resulted in a more comprehensive analysis (Fig. 1c). A linear trend with R^2^ = 0.36 and a normalized root-mean-square error (NRMSE) of 1.5 was observed between modification change (experimental data, y-axis) and the interface distances (native structures, x-axis) for labeled residues. A larger distance between a labeled residue and the other subunits in the bound form correlated with generally smaller changes in modification. This linear correlation observed was similar to previous work comparing solvent exposure metrics (solvent accessible surface area and neighbor count) and covalent labeling^36,38^. We used this correlation to predict an expected modification change from any structural model (by calculating the distances to the interface and using the fit line). The linear parameters of slope and intercept (Fig. 1c) were incorporated within our covalent labeling score term, as described in Methods.

### Structure prediction with covalent labeling data

RosettaDock has had many successes in modeling quaternary protein structure^58^. And its docking predictive capabilities can be further enhanced with the inclusion of sparse experimental data^59–62^. The benefit of using integrative modeling is that the results depend on the combination of Rosetta score and experimental data correlation, not one individually. The RosettaDock Interface score (Isc) accounts for interactions at the binding interface and can be supplemented with additional score terms to predict more nativelike poses. Here we aimed to explore whether covalent labeling MS data can meaningfully improve model quality. Due to the accuracy of AlphaFold2 (AF2) for monomer prediction, models generated by AF2 were used to provide the input to RosettaDock and a covalent labeling-based score term was used to rescore the oligomeric structures of modeled protein complexes and predict the native structure.

The parameters obtained from the correlation (Fig. 1c; a slope of −2.07 and an intercept of 46.27) were used to simulate predicted modification changes of labeled residues. For each labeled residue in a modeled complex, the interface distance was used to calculate a predicted modification change. Then the difference between experimentally observed and predicted modification change was calculated and input into a sigmoidal penalty term which penalized residues showing larger disagreement with experimental data (see Eq. 3 in Methods). The scores from the penalty function were then summed up for each labeled residue in a model and normalized across all models of a set. The resulting normalized score from the covalent labeling score term was then weighted and added to the Isc to form the covalent labeling score. Since traditional docking consists of two docking partners, the insulin complex was broken up into three separate sub-complexes to model the assembly of all unique interfaces, where AB_CD, ABCD_EFGH, ABCDEFGH_IJKL define what chains made up each docking partner, separated by an underscore. In a first study, we redocked the native crystal structures and Rosetta yielded accurate predictions for 4/5 complexes (Supplementary Fig. 2a). The only exception was β-2-microglobulin, for which a top-scoring model with an RMSD of 9.2 Å was identified. When including covalent labeling data in the score function, 5/5 complexes had accurate predictions and the top-scoring model for β-2-microglobulin had an RMSD of 3.0 Å (Supplementary Fig. 2b). While these data were promising, the preliminary docking study required crystallographic information of subunit structure in the complex state.

**Fig. 2:**
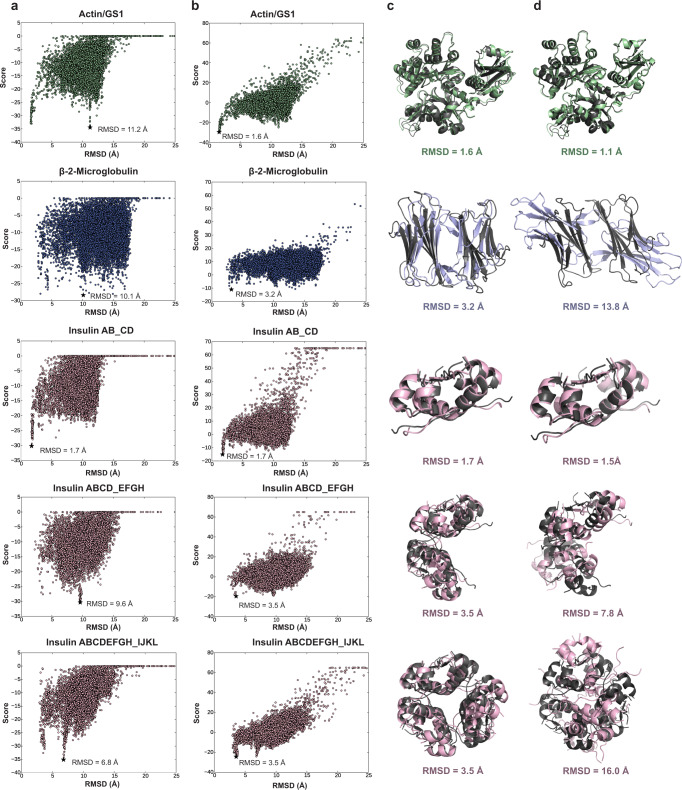
Score vs. RMSD to the crystal structure for 10,000 docked models generated for each complex in the benchmark set using AlphaFold2 models as docking input. The top-scoring models are marked by a black star and aligned to the crystal structure. Actin/gs1 is shown in green, β−2-microglobulin in blue, and insulin structures in pink. The RMSD of the top-scoring model is indicated next to the marked point. **a** RosettaDock Isc versus RMSD (without CL data). **b** Covalent labeling score versus RMSD (with CL data). Covalent labeling scores are less negative than Isc scores due to the addition of positive penalty scores. **c** Top-scoring complex model predictions using covalent labeling score aligned to the native crystal structures (dark gray). **d** Top-ranked models generated by AlphaFold-Multimer aligned to native crystal structure. RMSDs are listed for each complex. Source data are provided as a Source Data file.

To simulate a more realistic situation, we then used AF2 to generate components (subunits or sub-complexes) of the complexes, which were then input into docking simulations. The top-ranked AF2 models were all accurate with respect to the native structure with RMSD values of 1.2 Å and 0.7 Å for actin and gs1 A and G chains respectively, 1.6 Å for β-2-microglobulin chains, and 1.5 Å for insulin heterodimer. Scoring of the docked sets using covalent labeling data was performed by combining the covalent labeling score term produced by our method with Isc, as previously described. The score versus RMSD plots without using covalent labeling data are shown in Fig. 2a, where the top-scoring model RMSD with respect to the native structure was 11.2 Å for actin/gs1, 10.1 Å for β-2-microglobulin, 1.7 Å for insulin AB_CD, 9.6 Å for insulin ABCD_EFGH, and 6.8 Å for insulin ABCDEFGH_IJKL. Only 1/5 of the sets of docked structures had a top-scoring model with RMSD less than 5 Å. Figure 2b shows the results of docked sets from Fig. 2a using our covalent labeling score instead of Isc. Using our score, 5/5 of the sets had top-scoring models with an RMSD below 3.6 Å. The top-scoring model RMSD with respect to the native structure was 1.6 Å for actin/gs1, 3.17 Å for β-2-microglobulin, 1.73 Å for insulin AB_CD, 3.53 Å for insulin ABCD_EFGH, and 3.54 Å for insulin ABCDEFGH_IJKL. Figure 2c shows the top-scoring models for each docked set with the inclusion of our covalent labeling score term aligned to the native crystal structure.

The assessment of additional metrics further demonstrated the benefits of including covalent labeling in scoring. As shown in Table 1, improvements were observed in TM-score and DockQ score upon addition of CL data. TM-score analyzes the topological similarity between structures and DockQ is a quality measure used for evaluation of protein-protein docking data. The average TM-score improved from 0.70 to 0.84 (further improvement of high fold similarity) and the average DockQ score improved from 0.21 (an incorrect structure) to 0.50 (a medium quality structure) when including covalent labeling data in scoring. The TM-score and DockQ score for all top-scoring models either stayed the same or improved with the addition of experimental data (Supplementary Table 1). These results demonstrated that the information contained in the covalent labeling modification of residues can indeed facilitate the discrimination of nativelike and non-nativelike poses.

**Table 1:** Average metric analysis for the top-scoring models with and without covalent labeling data. Source data are provided as a Source Data file

	Avg RMSD (Å)	Avg TM-Score	Avg DockQ Score
w/o CL data	7.89	0.70	0.21
w/ CL data	2.70	0.84	0.50

As a comparison to state-of-the-art methodology, we also used AlphaFold-Multimer to predict the full structure of the complexes from our benchmark set without including the native structure as a homolog. Figure 2d shows the generated AlphaFold-Multimer models aligned to the native structures for the complexes. The root-mean-squared deviation (RMSD) of the top-ranked models for each of the complexes were 1.1 Å, 13.8 Å, 1.5 Å, 7.8 Å, and 16.0 Å for actin/gs1, β-2-microglobulin, insulin AB_CD, insulin ABCD_EFGH, and insulin ABCDEFGH_IJKL, respectively. Only 2/5 complexes in the benchmark set were accurately predicted with an RMSD below 7 Å. Interestingly, for the β-2-microglobulin homodimer, AlphaFold-Multimer predicted accurate individual chains in its top-ranked model (with an RMSD of 1.6 Å for both chains) but failed to accurately predict the full complex. This could be the result of loop regions (S11-N21 and F56-W59) present at the edges of the binding interface which may impede AlphaFold-Multimer’s ability to orient the subunits correctly. The inclusion of CL data (with labeled residues H13, K19, and K58 located in these loop regions) provides structural insights which may help overcome the incorrect predictions. It can be seen in Fig. 2d that the interface and orientations of the separate chains did not match that of the native structure.

## Conclusion

Sparse experimental data can bolster the effectiveness of existing computational techniques. In this current study, we have proposed a hybrid technique utilizing the combination of state-of-the-art computational methods (AlphaFold and RosettaDock) with covalent labeling mass spectrometry data to address cases when the computational tools fail to model accurate complexes. Covalent labeling reagents modify residues based on features such as solvent accessibility, and we have demonstrated that changes in modification of residues in covalent labeling experiments can be used to determine the likely proximity of these residues to the binding interface within protein complexes (Fig. 1). As the modification change of a labeled residue between the unbound/bound states of a complex increases, it is more likely to be located at the binding interface. The relationship between experimental modification change and inter-subunit distance was used to predict modification changes of modeled residues. We demonstrated that RosettaDock with the inclusion of our covalent labeling score term can predict accurate models for all the complexes in our benchmark set using AF unbound structures as input. Large improvements in model quality were observed when our score term was included. For example, the RMSD of the top-scoring model improved from 11.2 Å to 1.6 Å for actin/gs1 and 10.1 Å to 3.2 Å for β-2-microglobulin (Fig. 2a, b). This demonstrated that the information contained in the experimental covalent labeling values can improve scoring and model selection within RosettaDock. For protein systems with greater flexibility which are more likely to experience induced structural changes, this method may not be suitable due to our method’s assumption that large structural changes do not occur upon binding. This score term can be used through the newly developed cl_complex_rescore application within Rosetta. A tutorial for using this application can be found in Supplementary Note 1 within the Supplementary Information. Future work will include increasing the number, oligomeric state, and structural types of labeled proteins, along with the types of covalent labeling reagents used, to more comprehensively test the ability of covalent labeling data to elucidate protein complex structure. Additionally, the use of multiple orthogonal labeling techniques to study a single protein complex could be a promising avenue to potentially maximize the structural information obtained from covalent labeling experiments due to greater sequence and residue type coverage. For example, the simultaneous use of both HRF and DEPC/NHSA labeling yields a significantly greater coverage of the ‘optimal’ residue subset (6/9 of optimal set if combined, as discussed in methods) and total sequence coverage. In this study, we exclusively used differential covalent labeling data since it provides the most accurate structural information. However, many labeling experiments only yield non-differential datasets. In future work, we will focus on developing computational tools that utilize these datasets for complex prediction. In addition, we plan to explore combining other types of complementary experimental MS data with covalent labeling data.

## Methods

### Protein complex benchmark set

The three protein complexes used in the benchmark dataset were actin bound to gelsolin segment 1 (actin/gs1, heterodimer, PDB ID: 1YAG)^51^, β-2-microglobulin (homodimer, PDB ID: 2F8O)^52^, and insulin (hexamer of heterodimers, PDB ID: 4INS)^53^. Crystal structures were available for each for the purpose of benchmarking predicted models. Residue-resolved differential covalent labeling data were also obtained for each system in both the unbound and bound states^51–53^. The labeling reagent used for the actin/gs1 and insulin complexes was hydroxyl radicals and for β-2-microglobulin, the labeling reagents were diethyl pyrocarbonate (DEPC) and sulfo-*N*-hydroxysuccinimide (NHSA). We previously showed that labeling a subset of ‘optimal’ residues (G, R, K, L, T, F, S, V, and D) provides the highest amount of structural information useful in structure prediction^37^. HRF reliably labels L and F (2/9 of optimal set) and DEPC/NHSA labels R, K, T, and S (4/9 of optimal set). There were 41 labeled residues for both the unbound and bound states for actin/gs1, 20 for β-2-microglobulin, and 17 for insulin. For benchmarking purposes, interface residues were defined as any residue with a heavy atom within 10 Å of a heavy atom in another protein subunit. Although each labeled residue had a measure for the frequency of modification in both the unbound and bound states separately, we wanted to directly quantify the change in modification between these states, hypothesizing that residues with large changes would likely be part of the protein-protein interface. For each complex in the data set, the modification change between different states of the complex was computed from the data, as shown in Eq. 1, using the degree of labeling for each complex where ${{{{{{\rm{M}}}}}}}_{{{{{{\rm{unbound}}}}}}}$ and ${{{{{{\rm{M}}}}}}}_{{{{{{\rm{bound}}}}}}}$ are the degree of modification (modification rates for actin/gs1 and insulin, extent of modification for β-2-microglobulin) of the unbound and bound states of the complex, respectively.

1 $${Modification}\,{Change}=\frac{{M}_{{unbound}}-{M}_{{bound}}}{{M}_{{un}{bound}}}*100\%$$

### Protein-protein docking

Docking simulations require input subunit structures which are used to predict the structure of complexes. In this work, we obtained input structures using two different methods. First, we used the bound crystal structures to perform a preliminary redocking study. Next, structures for each docking partner of actin/gs1 and β-2-microglobulin were generated using AlphaFold2 (AF2) for a more realistic prediction protocol^20^. For insulin, the base subunit is a heterodimer, so AlphaFold-Multimer^24^ was used. The default settings for both AlphaFold methods were used along with the addition of all genetic databases (–db_preset=full_dbs flag). Since traditional docking consists of two docking partners, the insulin complex was broken up into three separate structures to model all unique interfaces, where AB_CD, ABCD_EFGH, ABCDEFGH_IJKL define what chains make up each docking partner, separated by an underscore (Supplementary Fig. 3). The docking protocol using RosettaDock was the same for each type of input structure. For each system, after prepacking, we generated sets of 10,000 docked models. The position and orientation of the second docking partner was randomized using the -randomize2 flag in the RosettaDock protocol to perturb each system.

### Complexes generated using AlphaFold-multimer

As a comparison to the docked models produced by RosettaDock, we also used AlphaFold-Multimer to predict full structures of each complex. To generate a more fair, blind prediction using AlphaFold-Multimer, restrictions were placed on which templates were used during model construction, as recommended by AlphaFold developers^20^. We modified the AlphaFold-Multimer input to only use PDB templates of structures that were deposited prior to the date of the first published structure of each complex to prevent any biased homology modeling based on existing crystal structures of the complexes.

### Scoring strategy

In this study, we proposed that differential covalent labeling data (comparing the unbound and bound states of a complex) could be used to indicate the proximity of a labeled residue to the binding interface of protein complexes and subsequently be used to assess model quality based on agreement with the experimental data. This was accomplished by comparing the modification change (Eq. 1) of labeled residues and the distance from the interface in the crystal structures. The interface distance (Fig. 1b) was defined as the shortest distance between a heavy atom of the target residue and a heavy atom from the binding partner. This comparison yielded an expected, inverse linear correlation between modification change and interface distance with the slope and intercept of the trendline being −2.07 and 46.27, respectively. The linear parameters of this trendline were used to predict modification changes of modeled residues for subsequent scoring based on comparisons to experimentally determined modification changes.

Therefore, to integrate the information regarding the modification change and interface distance into Rosetta to improve model scoring, a covalent labeling score term (${{{{{\rm{C}}}}}}{{{{{{\rm{L}}}}}}}_{{{{{{\rm{Score}}}}}}\_{{{{{\rm{Term}}}}}}}$) was developed to assess the models generated with RosettaDock based on their agreement or disagreement with covalent labeling data. The covalent labeling score (${{{{{\rm{C}}}}}}{{{{{{\rm{L}}}}}}}_{{{{{{\rm{Score}}}}}}}$), as defined in Eq. 2, was the sum between the ${{{{{\rm{C}}}}}}{{{{{{\rm{L}}}}}}}_{{{{{{\rm{Score}}}}}}\_{{{{{\rm{Term}}}}}}}$ (described in the following paragraph) and the Rosetta Interface score (Isc). The ${{{{{\rm{C}}}}}}{{{{{{\rm{L}}}}}}}_{{{{{{\rm{Score}}}}}}\_{{{{{\rm{Term}}}}}}}$ produced the best results within a weight range of 60–90, so a weight of 65 was chosen.

2 $${{{{{{\rm{CL}}}}}}}_{{{{{{\rm{Score}}}}}}}={{{{{\rm{Isc}}}}}}+65{{{{{{\rm{CL}}}}}}}_{{{{{{\rm{Score}}}}}}\_{{{{{\rm{Term}}}}}}}$$

3 $${{{{{\rm{C}}}}}}{{{{{{\rm{L}}}}}}}_{{{{{{\rm{Score}}}}}}\_{{{{{\rm{Term}}}}}}}=\mathop{\sum}\limits_{i}{P}_{i}=\mathop{\sum}\limits_{i}\left(1-\frac{1}{1+{e}^{A\left({d}_{i}-B\right)}}\right)$$

Isc was the energy of the binding interface of the docked complex calculated using the Rosetta REF2015 score function^31^. ${{{{{\rm{C}}}}}}{{{{{{\rm{L}}}}}}}_{{{{{{\rm{Score}}}}}}\_{{{{{\rm{Term}}}}}}}$, defined in Eq. 3, was a sum of per-residue penalties (${P}_{i}$) calculated using a sigmoidal penalty function. The penalty function scores labeled residues of a model based off deviations of predicted modification changes and modeled interface distances from the observed trendline of the native dataset (with large deviations from experimental results penalized).

For each labeled residue of a given model, interface distance is calculated and used to predict modification change using the slope and intercept defined above. The difference $({d}_{i})$ between the experimental and predicted modification change was input into the penalty function. The A and B parameters defined the steepness and midpoint of the curve respectively, where *A* = 1.88 and *B* = 38.0. The summed penalties for all models are then normalized by dividing each score by the maximum score obtained for that particular system. Thus, the resulting ${{{{{\rm{C}}}}}}{{{{{{\rm{L}}}}}}}_{{{{{{\rm{Score}}}}}}\_{{{{{\rm{Term}}}}}}}$ ranges from 0 to 1 where greater deviation from the trendline (indicating worse agreement with the experimental data) results in a larger penalty score from the score term.

### Analysis metrics

The quality of models was assessed quantitively using alpha-carbon root-mean-squared deviation (RMSD), template modeling score (TM-score)^63^, and DockQ^64^ score with respect to the native crystal structure. For each model, the global RMSD values were calculated using PyMol^65^. TM-score was used to analyze the topological similarity to the native structures. The TM-score ranges from 0.0 to 1.0 where a perfect match corresponds to a TM-score of 1.0. TM-score classifies models as either having random structural similarity (0.0 <TM-score <0.17) or high fold similarity (0.5 <TM-score <1.00) to the native structure. DockQ is a protein-protein docking quality measure which ranges between 0.0 and 1.0 with a perfect match being equal to 1.0. Similar to TM-score, DockQ uses four categories for classifying models: incorrect (0 <DockQ score <0.23), acceptable quality (0.23 < = DockQ score <0.49), medium quality (0.49 < = DockQ <0.80), and high quality (DockQ score > 0.80).

### Software usage for data analysis

Python v.3.7.3 was used for data analysis. Matplotlib v.3.1.2 was used for the creation of all scatter plots. PyMol v.2.0.7 was used to generate the figures of all proteins.

### Reporting summary

Further information on research design is available in the Nature Portfolio Reporting Summary linked to this article.

## Supplementary information

Supplementary Information

Reporting Summary

Description of Additional Supplementary Files

Supplementary Data 1

## Data Availability

The crystal structure data used in this study were obtained from the Protein Data Bank (https://www.rcsb.org) with accession codes1YAG, 2F8O, 4INS. The accession codes of the unbound structures are 3HBT, 2D4F, and 3I40. A subset of 200 docked models for each structure generated using AlphaFold and RosettaDock (including the 100 top-scoring models before and after using CL data) as well as the labeling data used in this work are available in the Supplementary Information as Supplementary Data 1. Access to the complete set of models (not available due to size limitations) can be obtained by emailing the corresponding author (lindert.1@osu.edu). Source data are provided with this paper.

## Source data

Source Data

## Code availability

The cl_complex_rescore application is available for free to academic users through the Rosetta software suite at https://www.rosettacommons.org/software/. The current academic version of Rosetta (3.13) can be freely downloaded from https://els2.comotion.uw.edu/product/rosetta for academic users. The source code for the cl_complex_rescore application (which is part of the Rosetta codebase) is only made available to academic/non-profit/government entities and commercial entities with a Company Contributor License. While availability to the Rosetta codebase is free for academics/non-profit/government entities, note that there is a Rosetta license fee for industry users to gain access to the source code and the applications in Rosetta (including the cl_complex_rescore application). Currently the University of Washington exclusively manages all Rosetta licensing. More information on Rosetta licensing can be found at https://www.rosettacommons.org/about/faq. Instructions to run the cl_complex_rescore application in Rosetta can be found in Supplementary Note 1 within the Supplementary Information.
