# Supplementary material for: Protein complex prediction using Rosetta, AlphaFold, and mass spectrometry covalent labeling
Source: Nat Commun. 2022 Dec 21;13:7846. doi: 10.1038/s41467-022-35593-8 (PMC9772387; doi:10.1038/s41467-022-35593-8)
Supplement: Supplementary file 2 — Reporting Summary [file 41467_2022_35593_MOESM2_ESM.pdf]

nature portfolio

Corresponding author(s): Steffen Lindert
Last updated by author(s): Nov 25, 2022

Reporting Summary

Nature Portfolio wishes to improve the reproducibility of the work that we publish. This form provides structure for consistency and transparency in reporting. For further information on Nature Portfolio policies, see our Editorial Policies and the Editorial Policy Checklist.

Statistics

For all statistical analyses, confirm that the following items are present in the figure legend, table legend, main text, or Methods section.

n/a	Confirmed	
☐	☒	The exact sample size (*n*) for each experimental group/condition, given as a discrete number and unit of measurement
☒	☐	A statement on whether measurements were taken from distinct samples or whether the same sample was measured repeatedly
☒	☐	The statistical test(s) used AND whether they are one- or two-sided<br>*Only common tests should be described solely by name; describe more complex techniques in the Methods section.*
☒	☐	A description of all covariates tested
☒	☐	A description of any assumptions or corrections, such as tests of normality and adjustment for multiple comparisons
☒	☐	A full description of the statistical parameters including central tendency (e.g. means) or other basic estimates (e.g. regression coefficient) AND variation (e.g. standard deviation) or associated estimates of uncertainty (e.g. confidence intervals)
☒	☐	For null hypothesis testing, the test statistic (e.g. *F*, *t*, *r*) with confidence intervals, effect sizes, degrees of freedom and *P* value noted<br>*Give P values as exact values whenever suitable.*
☒	☐	For Bayesian analysis, information on the choice of priors and Markov chain Monte Carlo settings
☒	☐	For hierarchical and complex designs, identification of the appropriate level for tests and full reporting of outcomes
☒	☐	Estimates of effect sizes (e.g. Cohen's *d*, Pearson's *r*), indicating how they were calculated

Our web collection on statistics for biologists contains articles on many of the points above.

Software and code

Policy information about availability of computer code

Data collection	Alpha Fold v2.0.0 (https://github.com/deepmind/alphafold/releases/tag/v2.0.0) for protein monomeric structure prediction. The cl_complex_rescore application for rescoring with CL data is freely available to academic users through the Rosetta Software Suite (v.3.13) at https://www.rosettacommons.org/software.
Data analysis	Python v.3. 7.3 (https://www.python.org/downloads/release/python-373/), Matplotlib v.3.1.2 (https://matplotlib.org/3.1.1/users/installing.html), PyMOL v.2.0.7 (https://pymol.org/installers/), DockQ v.1.0 (https://github.com/bjornwallner/DockQ)

For manuscripts utilizing custom algorithms or software that are central to the research but not yet described in published literature, software must be made available to editors and reviewers. We strongly encourage code deposition in a community repository (e.g. GitHub). See the Nature Portfolio guidelines for submitting code & software for further information.

Data

Policy information about availability of data

All manuscripts must include a data availability statement. This statement should provide the following information, where applicable:

- Accession codes, unique identifiers, or web links for publicly available datasets
- A description of any restrictions on data availability
- For clinical datasets or third party data, please ensure that the statement adheres to our policy

The Protein Data Bank (PDB) was used for all the crystal structure used in this study. The accession codes of the complexes used in the dataset are: 1YAG, 2F8O,

4INS. The accession codes of the unbound structures are 3HBT, 2D4F, and 3I40. These structures can be accessed from the PDB (https://www.rcsb.org) with the accession codes provided here. The models generated using AlphaFold and RosettaDock as well as the labeling data used in this work are available in the Supplementary Information as a zipped file. Source data are provided with this paper.

Human research participants

Policy information about studies involving human research participants and Sex and Gender in Research.

Reporting on sex and gender	N/A
Population characteristics	N/A
Recruitment	N/A
Ethics oversight	N/A

Note that full information on the approval of the study protocol must also be provided in the manuscript.

Field-specific reporting

Please select the one below that is the best fit for your research. If you are not sure, read the appropriate sections before making your selection.

☒ Life sciences ☐ Behavioural & social sciences ☐ Ecological, evolutionary & environmental sciences

For a reference copy of the document with all sections, see nature.com/documents/nr-reporting-summary-flat.pdf

Life sciences study design

All studies must disclose on these points even when the disclosure is negative.

Sample size	For the experimental dataset, the sample size was determined based on the availability of previously published CL data in the literature. The sample size for docked model generation was chosen to be 10,000 based on the rationale that given the size of the proteins, this is a large enough number for Monte Carlo sampling.
Data exclusions	No data were excluded.
Replication	Since Rosetta employs a Monte Carlo modeling algorithm, the sampling distribution may not be exactly identically reproduced. But the overall distribution should still more or less be the same. The actual rescoring is reproducible once docked models have been generated. We have provided a set of 200 docked models (zipped) for Actin/GS1 (1YAG PDB-ID), β-2-microglobulin (2F8O PDB-ID), and Insulin (4INS PDB-ID) complexes where monomeric subunits were generated using AlphaFold2. Users can test reproducibility on this data subset.
Randomization	Not applicable; no sample/organism/participant experimental groups were used.
Blinding	Not applicable; no sample/organism/participant experimental groups were used.

Reporting for specific materials, systems and methods

We require information from authors about some types of materials, experimental systems and methods used in many studies. Here, indicate whether each material, system or method listed is relevant to your study. If you are not sure if a list item applies to your research, read the appropriate section before selecting a response.

Materials & experimental systems

n/a	Involved in the study
☒	☐ Antibodies
☒	☐ Eukaryotic cell lines
☒	☐ Palaeontology and archaeology
☒	☐ Animals and other organisms
☒	☐ Clinical data
☒	☐ Dual use research of concern

Methods

n/a	Involved in the study
☒	☐ ChIP-seq
☒	☐ Flow cytometry
☒	☐ MRI-based neuroimaging
