# Supplementary material for: Protein complex prediction using Rosetta, AlphaFold, and mass spectrometry covalent labeling
Source: Nat Commun. 2022 Dec 21;13:7846. doi: 10.1038/s41467-022-35593-8 (PMC9772387; doi:10.1038/s41467-022-35593-8)
Supplement: Supplementary file 3 — Description of Additional Supplementary Files [file 41467_2022_35593_MOESM3_ESM.pdf]

File name: Supplementary Data 1

Description: A subset of 200 docked models for each structure generated using AlphaFold and RosettaDock (including the 100 top-scoring models before and after using CL data) as well as the labeling data used in this work.
